# The Association of Plant-Based Diet With Cardiovascular Disease and Mortality: A Meta-Analysis and Systematic Review of Prospect Cohort Studies

**DOI:** 10.3389/fcvm.2021.756810

**Published:** 2021-11-05

**Authors:** Jingxuan Quek, Grace Lim, Wen Hui Lim, Cheng Han Ng, Wei Zheng So, Jonathan Toh, Xin Hui Pan, Yip Han Chin, Mark D. Muthiah, Siew Pang Chan, Roger S. Y. Foo, James Yip, Nithya Neelakantan, Mary F. F. Chong, Poay Huan Loh, Nicholas W. S. Chew

**Affiliations:** ^1^Yong Loo Lin School of Medicine, National University of Singapore, Singapore, Singapore; ^2^Lee Kong Chian School of Medicine, Nanyang Technological University, Singapore, Singapore; ^3^Division of Gastroenterology and Hepatology, Department of Medicine, National University Hospital, Singapore, Singapore; ^4^National University Centre for Organ Transplantation, National University Health System, Singapore, Singapore; ^5^Department of Cardiology, National University Heart Centre, National University Hospital, Singapore, Singapore; ^6^Saw Swee Hock School of Public Health, National University Health System, National University of Singapore, Singapore, Singapore

**Keywords:** vegetarians, vegans, heart disease, cardiovascular disease, plant-based diets

## Abstract

**Background:** The association between plant-based diets and cardiovascular disease (CVD) remains poorly characterized. Given that diet represents an important and a modifiable risk factor, this study aimed to assess (1) the relationships between the impact of adherence to plant-based diets on cardiovascular mortality, incident CVD, and stroke; (2) if associations differed by adherence to healthful and less healthful plant-based diets.

**Methods and Findings:** MEDLINE and EMBASE databases were searched up to May 2021. Studies assessing CVD outcomes with relation to plant-based dietary patterns or according to plant-based dietary indices (PDI) were included. A meta-analysis of hazard ratios (HR) was conducted using DerSimonian and Laird random effects model. Thirteen studies involving 410,085 participants were included. Greater adherence to an overall plant-based dietary pattern was significantly associated with a lower risk of cardiovascular mortality (pooled HR: 0.92, 95% CI: 0.86–0.99 *p* = 0.0193, *I*^2^ = 88.5%, *N* = 124,501) and a lower risk of CVD incidence (pooled HR: 0.90, 95% CI: 0.82–0.98, *p* = 0.0173, *I*^2^ = 87.2%, *N* = 323,854). Among the studies that used PDI, unhealthful plant-based diets were associated with increased risk of cardiovascular mortality (pooled HR: 1.05, 95% CI: 1.01–1.09, *p* = 0.0123, *I*^2^ = 0.00%, *N* = 18,966), but not CVD incidence. Conversely, healthful plant-based diets were associated with decreased CVD incidence (pooled HR: 0.87, 95% CI: 0.80–0.95, *p* = 0.0011, *I*^2^ = 57.5%, *N* = 71,301), but not mortality. Vegetarians also had significantly lower CVD incidence (HR: 0.81, 95% CI: 0.72–0.91, *p* = 0.0004, *I*^2^ = 22.2%, *N* = 16,254), but similar CVD mortality or stroke risk when compared to the meat-eaters.

**Conclusion:** To date, this comprehensive study examines the effects of a plant-based diet on major clinical endpoints using more holistic PDIs. These findings highlight the favorable role of healthful plant-based foods in reducing cardiovascular mortality and CVD.

## Introduction

Cardiovascular disease (CVD) is the leading cause of death, accounting for 32% of all global deaths in 2019 (1). In 2016, 3,63,452 and 1,42,142 individuals died from ischemic heart disease and stroke, respectively in the United States alone (2). Given the significant mortality and morbidity, diet represents an important modifiable risk factor that impacts other cardiovascular risk factors in the management of CVD.

Growing evidence suggests potential cardiovascular benefits of plant-based diets and dietary patterns (3), defined as a dietary profile, which emphasizes the high intake of plant-based food products while limiting the intake of animal products. Higher consumption of plant foods has been shown to reduce systolic blood pressure and plasma triglyceride levels, thereby exerting protective effects against obesity and incident diabetes (4–7). Besides reducing CVD risk factors, previous studies have also indicated an association between plant-based diets and improved quality of life, such as an improved quality of sleep, reduced likelihood of mental health disorders, and decreased rate of cognitive decline (8–10).

Current literature on the association between a plant-based diet with major CVD clinical endpoints remain poorly characterized as prior studies have defined vegetarian or vegan diets based on the exclusion of animal food consumption (11–13). In 2012, Huang *et al*. found that vegetarians had a 29% lower ischemic heart disease associated mortality rate as compared to non-vegetarians (14). In contrast, a more recent study in 2019 revealed no significant association between vegetarian dietary pattern and CVD or stroke mortality (15), while another study in 2020 suggested that plant-based diets did not have a significant impact on CVD mortality amongst patients with diabetes (16), yielding conflicting results. This classification method fails to account for the opposing health effects of various plant-based food groups (17, 18), such as unhealthful refined grains, starches, and sugars as opposed to whole grains, fruits, and vegetables. However, there have since been new developments in plant-based diet scores with more studies utilizing the plant-based dietary index (PDI) where dietary scores are calculated based on gradations of adherence to a predominantly plant-based diet. Furthermore, variations such as the healthful and unhealthful PDI allow holistic assessment of the synergistic effects within dietary compositions (19, 20). Additionally, previous literature reported survival outcomes in risk ratio which does not account for censored data (14, 21–23), instead of hazard ratio which has been heralded as the gold standard approach in time-dependent survival analysis (24). Thus, this meta-analysis aimed to consolidate updated evidence and provide a more robust estimate of the overall effect of adherence to a plant-based diet on cardiovascular mortality, CVD, and stroke outcomes in hazard ratio. Additionally, this study also sought to assess if associations differed by adherence to healthful and less healthful plant-based diets based on *a priori* defined PDIs.

## Methods

### Search Strategy

This review was registered with PROSPERO (CRD42021265684) with adherence to the Preferred Reporting Items for Systematic Review and Meta-Analyses 2020 for its synthesis (25). Two electronic databases, MEDLINE and EMBASE, were searched for articles relating to plant-based diets and cardiovascular health outcomes from inception to May 30, 2021. Search terms included “plant-based diet,” “vegetarian,” “vegan,” its related synonyms, “cardiovascular mortality,” and “cardiovascular diseases” as outcome terms. The full search strategy can be found in the Supplementary File 1. All references were imported into Endnote X9 for duplicate removal. The references of the included articles were also manually screened to ensure all relevant articles were included.

### Eligibility and Selection Criteria

Two authors (JXQ and GL) independently performed the title abstract sieve and full-text review based on the predefined inclusion criteria. Any discrepancies were resolved by consensus or in consultation with a third independent author (NWSC). Only original articles in the English language were included, excluding conference abstracts, reviews, commentaries, and editorials. The primary exposure of interest was adherence to plant-based dietary patterns, defined as higher consumption of plant-based foods and lower consumption or exclusion of animal-based foods. In line with this definition, vegetarian or vegan dietary patterns were also classified under plant-based dietary patterns. Thus, the inclusion criteria were prospective cohort studies involving vegan or vegetarian participants or studies that assessed the plant-based diet of the subjects using various PDIs such as healthful PDI (hPDI) and unhealthful PDI (uPDI) with reported outcomes on cardiovascular mortality, cardiovascular disease, or stroke events. The exclusion criteria included (1) studies that focused on single food groups such as vegetables, beans, nuts, or specific cruciferous vegetables that did not reflect a major part of the diet; (2) studies that used *a priori* indices such as healthy eating index, alternate Mediterranean diet score, and dietary approaches to stop hypertension; and (3) studies which did not provide outcomes of interests in the hazard ratio (HR).

### Data Extraction

Relevant data from included articles were extracted by a pair of independent authors (JXQ and GL) into a structured proforma. The primary outcomes of interest were cardiovascular mortality, CVD, and stroke. Cardiovascular mortality was defined in accordance to the International Classification of Diseases (ICD), 9th or 10th edition (26, 27), whereas CVD was defined as the composite endpoint of coronary heart disease including non-fatal or fatal myocardial infarction, heart failure defined as hospitalization or death with reference to ICD-9 code 428 and ICD-10 code I50, or stroke including definite or probable stroke. Stroke was defined according to (1) the World Health Organisation (WHO) as focal neurological deficit lasting >24 h or non-focal neurological symptoms with imaging consistent of stroke (28), or (2) identified based on the ICD-8, 9, or 10 codes including total, ischemic, or haemorrhagic stroke. Study characteristics were also extracted, including author, year, country, study design, follow-up duration, sample size, patient demographics i.e., age, gender, body mass index (BMI), alcohol history, smoking history, dietary assessment method, type of PDI, level of intake category (e.g., tertile, quantile, or quintile), and covariates that were adjusted for in the statistical models. For studies that used dietary indices, hazard estimates comparing the best (highest quintile/quantile) and poorest (lowest quintile/quantile) adherence to the plant-based dietary pattern were extracted. For studies that compared plant-based dietary patterns, the authors extracted the hazard estimates which compared diets that are most restrictive of animal-based foods such as vegan or vegetarian diets with the least restrictive, such as regular meat-eaters. All extracted information were checked independently by both authors to ensure accuracy of data collection with any discrepancies resolved through further discussion to reach a consensus.

### Statistical Analysis and Quality Assessment

All analysis was done in R studio (Version 1.3.1,093) using the meta package. A conventional pairwise meta-analysis was conducted in hazard ratio using the DerSimonian and Laird random effects model to assess the association of plant-based diets with cardiovascular health outcomes (29). The risk estimate with the greatest degree of statistical adjustment was included in the meta-analysis. Statistical heterogeneity was assessed with the *I*^2^ statistic and the Cochran *Q* test, where a *p*-value of ≤ 0.10 was considered significant for heterogeneity (30, 31). An *I*^2^ value of 25, 50, and 75% represented low, moderate, and a high degree of heterogeneity, respectively (30). The random effects model was used in all analyses regardless of heterogeneity as recent evidence suggests that it can provide more robust outcome measures in comparison to the fixed effects model (32). However, *I*^2^ may be an inferior estimate when the sample size is large, leading to falsely elevated levels of heterogeneity (33, 34).

To explore potential sources of heterogeneity, the analysis was stratified according to definitions of plant-based diets i.e., vegetarian diet vs. plant-based dietary scores. In studies that classified adherence to plant-based dietary patterns using overall PDI, subgroup comparison was conducted for hPDI vs. uPDI to analyze the effects of quality of plant-based foods on cardiovascular health. Publication bias was assessed by visual inspection of the respective funnel plots (35). For quality assessment of the included studies, the Newcastle-Ottawa Quality Assessment Scale (NOS) for cohort studies was utilized to assess the risk of bias based on several parameters including appropriateness of sample frame, sampling method, ascertainment of exposure, the demonstration that outcome of interest was not present at the start of the study, comparability of cohorts, methods for assessment of outcomes, duration of follow-up, and adequacy of follow-up (36).

## Results

### Summary of Included Articles

**Figure 1:**
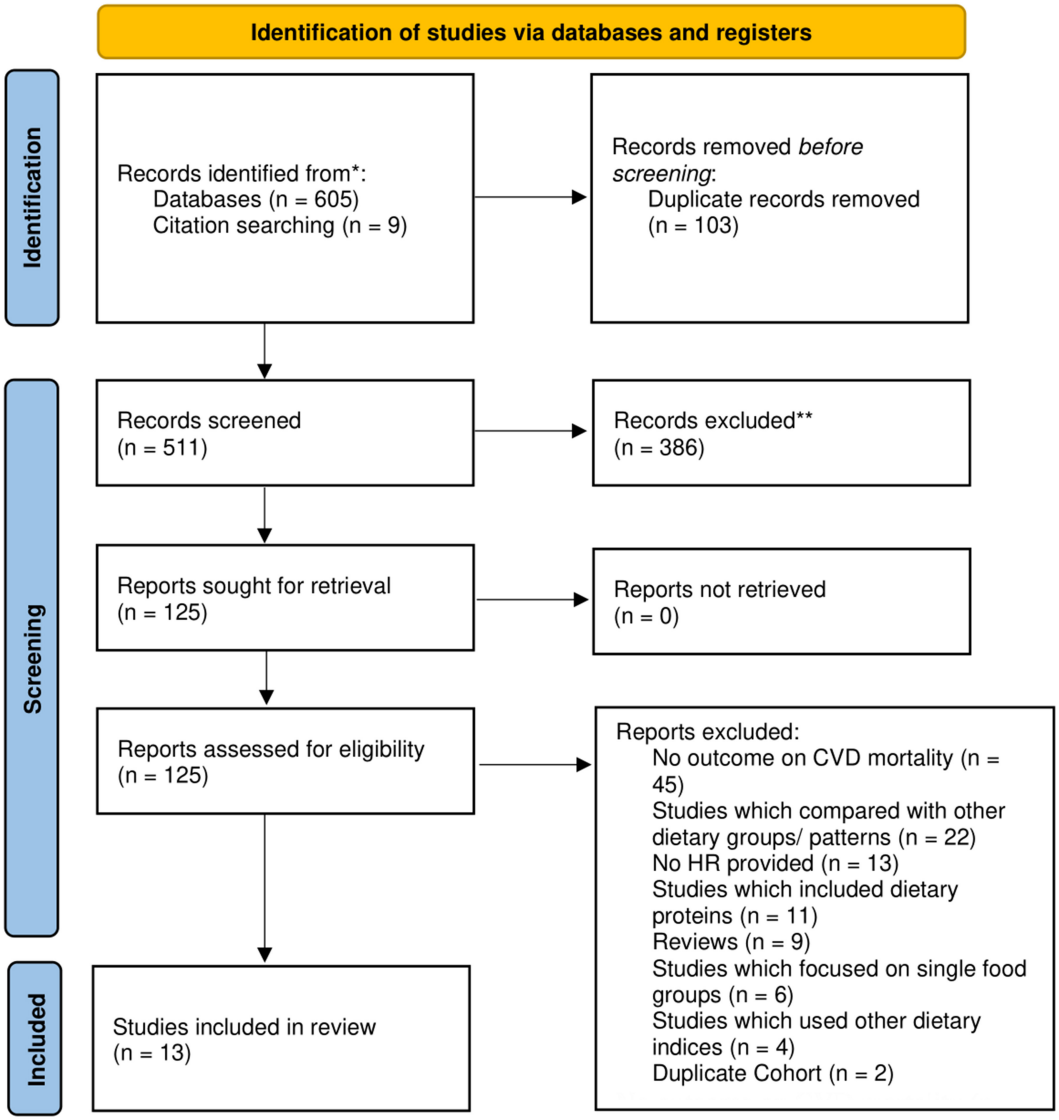
PRISMA flow diagram.

A systematic search of the literature yielded a total of 605 references before 103 duplicates were removed, following which 386 articles were excluded after the title abstract sieve. In addition, nine studies were retrieved for full text review after screening the references of included articles and other meta-analyses (14, 21, 22, 37–41). A total of 125 studies were selected for full text review, of which 13 studies were included in this meta-analysis (Figure 1). In total, 410,085 participants were included in our analysis, and 78,671 subjects were identified as vegetarian or vegan. Eight articles originated from North America (11, 42–48), four articles from Europe (12, 20, 49, 50), and one article from Asia (51). Table 1 summarizes the baseline characteristics and quality assessment of the included studies.

**Table 1:** Summary of included articles.

**Author**	**Year**	**Region**	**Sample Size**	**Mean Age**	**Gender (male)**	**BodyMass Index**	**Smoking History**	**Dietary Assessment Method**	**Exposure**	**Level of Intake Category**	Adjustments^*^															**Quality Assessment**
											**1**	**2**	**3**	**4**	**5**	**6**	**7**	**8**	**9**	**10**	**11**	**12**	**13**	**14**	**15**	
Heianza et al.	2020	Europe	30,410	56.8	0.358	26.0	0.54	Oxford WebQ	hPDI	Quintile	✓	✓	✓	✓	✓	✓	✓	✓	✓	✓	✓	✓	✓	✓	✓	7
Shan et al.	2020	North America	248,029	45.9	-	24.2	0.479	FFQ	PDI, hPDI, uPDI	Quintile	✓				✓	✓	✓	✓	✓	✓		✓	✓	✓	✓	7
Chiu et al.	2020	Asia	4,143	51.4	0.269	23.0	0.0962	FFQ, Interview	Vegetarian	-	✓	✓		✓		✓	✓			✓		✓	✓	✓	✓	8
Baden et al.	2019	North America	5,128	63	-	25	0.53	FFQ	PDI, hPDI, uPDI	Quintile	✓		✓		✓	✓	✓	✓		✓			✓	✓	✓	7
Kim et al.	2019	North America	8,740	38.9	0.596	-	0.152	FFQ, Interview	PDI, hPDI, uPDI, Provegetarian	Quintile	✓	✓	✓	✓		✓	✓			✓		✓	✓		✓	8
Lara et al.	2019	North America	16,068	64.9	0.339	28.6	0.074	FFQ	Plant-based	Quartile	✓	✓	✓	✓		✓	✓					✓	✓	✓		6
Tong et al.	2019	Europe	16,254	39.4	0.247	23.0	0.104	FFQ	Vegetarian	-	✓	✓		✓		✓	✓			✓	✓					8
Kim et al.	2018	North America	11,879	48	0.48	-	0.15	FFQ, Interview	PDI, hPDI, uPDI	Decile	✓	✓	✓	✓		✓	✓		✓	✓		✓	✓	✓		8
Appleby et al.	2016	Europe	20,324	43.4	0.247	23.5	0.165	FFQ	Vegan	-						✓	✓	✓		✓		✓	✓		✓	7
Shikany et al.	2015	North America	4,353	-	0.336	27.8	0.073	FFQ	Plant-based	Quartile	✓	✓	✓	✓		✓	✓		✓			✓	✓	✓	✓	6
Martinez-Gonzalez et al.	2014	Europe	1,731	67.4	0.424	29.7	0.118	FFQ, Interview	Provegetarian	-	✓	✓		✓		✓	✓		✓	✓						8
Judd et al.	2013	North America	5,076	-	0.37	-	0.07	FFQ	Plant- based	Quartile	✓	✓	✓	✓		✓			✓							6
Orlich et al.	2013	North America	37,950	57.8	0.344	25.9	0.138	FFQ	Vegetarian	-	✓			✓		✓	✓			✓						7

* *Legend, Quality Assessment was conducted with the Newcastle-Ottawa Scale for Cohort Studies; PDI, Plant-based Dietary Index; hPDI, Healthful Plant-based Dietary Index; uPDI, Unhealthful Plant-based Dietary Index. 1. Age, 2. Sex, 3. Ethnicity/Race, 4. Education, 5. Family history of heart disease, 6. Smoking habit, 7. Physical activity, 8. Multivitamin use, 9. Total energy intake, 10. Alcohol, 11. Townsend Deprivation Index, 12. Body Mass Index, 13. Hypertension, 14. Dyslipidaemia, 15. Type 2 diabetes. ✓The symbol denotes that the variable is reported in the included study*.

Included studies were prospective cohort studies with the mean age of the participants ranging from 38 to 67 years and mean BMI ranging from 23.0 to 29.7 kg/m^2^. The majority of the studies administered food frequency questionnaires to assess the dietary intake of participants (11, 20, 42–48, 50, 51), out of which four studies involved trained interviewers (20, 44, 46, 51). Three studies derived the plant-based dietary patterns of participants using a factor analysis approach (45, 47, 48) compared with the six studies which calculated plant-based dietary scores using PDI (42–44, 46, 49, 52). In total, four studies characterized adherence to plant-based dietary patterns using overall PDI (43, 44, 46, 52), four studies used hPDI (42, 44, 46, 49), four studies used uPDI (43, 44, 46, 52), and two studies used provegetarian food pattern (20, 44). In addition, four studies compared individuals following a vegetarian dietary pattern with regular meat eaters (11, 12, 50, 51). The majority of included studies were found to have a low risk of bias (*n* = 10) (11, 12, 20, 42–44, 46, 49, 51) whereas the remaining studies were at moderate risk of bias (*n* = 3) (45, 47, 48).

### Overall Analysis

The pooled analysis of seven studies involving 1,24,501 and 3,23,854 subjects demonstrated that a greater adherence to an overall plant-based dietary pattern was significantly associated with decreased risk of cardiovascular mortality (pooled HR: 0.92, 95% CI: 0.86–0.99, *p* = 0.0193, I^2^ = 88.5%, *N* = 124,501) and lower risk of CVD incidence (pooled HR: 0.90, 95% CI: 0.82– 0.98, *p* = 0.0173, *I*^2^ = 87.2%, *N* = 323,854), respectively. However, there was a non-significant decrease in the risks of total stroke with greater adherence to an overall plant-based diet (pooled HR: 0.86, CI: 0.69 - 1.08, *p* = 0.190, I^2^ = 79.1%, *N* = 64,204). However, there was no evidence of publication bias from the funnel plots in the Supplementary File 2.

### Plant-Based Dietary Index

#### Cardiovascular Mortality

**Figure 2:**
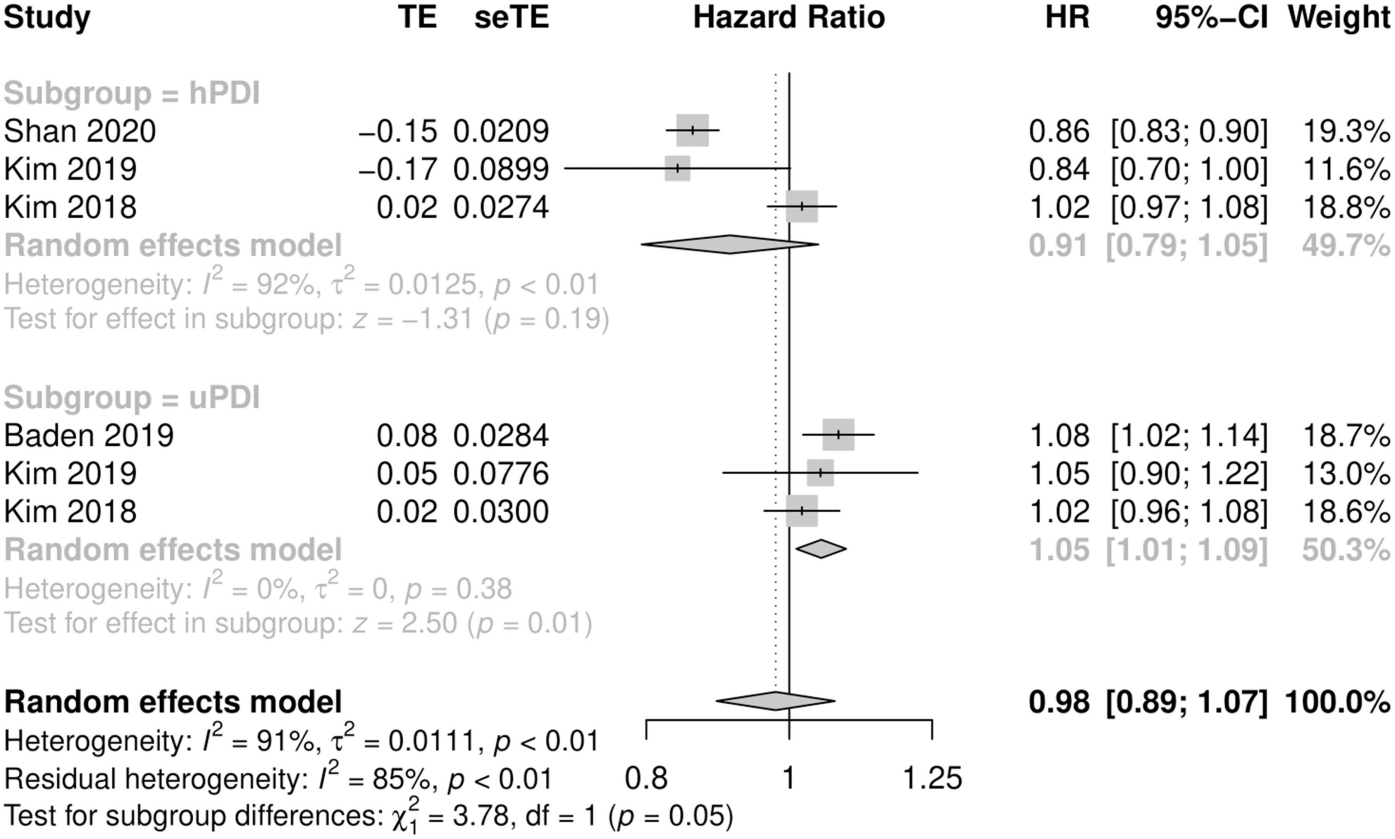
Cardiovascular mortality by healthful PDI (hPDI) and unhealthful PDI.

When results were stratified by studies (*n* = 7) that assessed plant-based diets using overall PDI, there was a non-significant decrease in cardiovascular mortality between subjects with the greatest adherence to a plant-based diet in the highest quintile compared with the least adherent subjects in the lowest quintile (pooled HR: 0.90, 95% CI: 0.76–1.05, *p* = 0.184, *I*^2^ = 91.7%, *N* = 28,844). Similarly, among studies using hPDI to calculate diet scores, cardiovascular mortality was found to be comparable between those in the highest and lowest quintile (pooled HR: 0.91, 95% CI: 0.79–1.05, *p* = 0.190, *I*^2^ = 92.1%, *N* = 52,770). In contrast, studies that utilized uPDI showed a significantly increased risk of cardiovascular mortality for subjects who adhered most closely to an unhealthful diet as compared with the least adherent subjects (pooled HR: 1.05, CI: 1.01–1.09, *p* = 0.0123, *I*^2^ = 0.00%, *N* = 18,966). There was a significant difference between hPDI and uPDI (*p* = 0.05) (Figure 2). Heterogeneity was high with *I*^2^ of 91.7 and 92.1% for overall PDI and hPDI, respectively, in contrast to uPDI which yielded low heterogeneity with *I*^2^ of 0.00%.

#### Cardiovascular Disease

Pooled analysis of seven studies using overall PDI showed that subjects with the greatest adherence to a plant-based diet had significantly lower risks of CVD compared with those who were the least adherent (pooled HR: 0.88, 95% CI: 0.81–0.96, *p* = 0.0054, *I*^2^ = 34.2%, *N* = 211,585). For studies using hPDI to calculate diet scores, subjects who were the most adherent to a healthful plant-based diet also had a decreased risk of CVD compared with those who were the least adherent (pooled HR: 0.87, 95% CI: 0.80–0.95, *p* = 0.0011, *I*^2^ = 57.5%, *N* = 71,301). However, analysis of studies utilizing uPDI showed no significant association between CVD and adherence of participants to an unhealthful plant-based diet (pooled HR: 1.11, 95% CI: 0.79–1.56, *p* = 0.555, *I*^2^ = 95.5%, *N* = 2,21,177). There was no significant subgroup difference between studies utilizing hPDI compared with uPDI (*p*-value: 0.18). There was low, moderate, and high heterogeneity with I^2^ of 34.2, 57.5, and 95.5% in overall PDI, hPDI, and uPDI analysis, respectively.

### Vegetarian Only

Participants following a vegetarian diet had a non-significant decrease in cardiovascular mortality compared with regular meat eaters (pooled HR: 0.89, 95% CI: 0.78–1.01, *p* = 0.0611, I^2^ = 0.00%, *N* = 58,274). However, vegetarians were found to have significantly reduced risks of CVD compared with regular meat eaters (pooled HR: 0.81, 95% CI: 0.72 - 0.91, *p* = 0.0004, I^2^ = 22.2%, *N* = 16,254). There was low heterogeneity for both cardiovascular mortality and CVD analysis with *I*^2^ of 0.00 and 22.2%, respectively. Further analysis showed a non-significant decreased risk of total stroke (pooled HR: 0.72, 95% CI: 0.36–1.41, *p* = 0.333, I^2^ = 81.9% *N* = 20,397), hemorrhagic stroke (pooled HR: 0.77, 95% CI: 0.19–3.09, *p* = 0.709, I^2^ = 84.8%, *N* = 18,973) and ischemic stroke (pooled HR: 0.56, 95% CI: 0.22–1.42, *p* = 0.219, I2 = 81.9%, *N* = 20,397) between vegetarians and regular meat eaters. There were high levels of heterogeneity with *I*^2^ of 87.3, 84.8, and 81.9% for total stroke, hemorrhagic stroke, and ischemic stroke, respectively.

## Discussion

To our knowledge, the present study provides comprehensive evidence on the associations between plant-based diets and major clinical endpoints including cardiovascular mortality, risk of CVD, and stroke. Previous analyses largely focused on vegetarian cohorts or proximal determinants of CVD and failed to clearly define the impact of adherence to plant-based dietary patterns on key clinical outcomes (11–15, 17, 18). However, in these meta-analyses of 410,085 individuals, we found that greater adherence to an overall plant-based dietary pattern significantly reduced the risks of cardiovascular mortality and development of CVD by 8.1% and 10.2%, respectively. Healthful plant-based diets including higher intake of whole grains, fruits, vegetables, nuts, legumes, tea, and coffee were found to have protective effects against CVD, whereas unhealthful plant-based diets were found to increase cardiovascular mortality (Table 2).

**Table 2:** Summary of cardiovascular mortality and cardiovascular disease outcomes.

	**Cardiovascular Mortality**						**Cardiovascular Disease**					
	**Number of Studies**	**Number of Patients**	**HR (95% CI)**	**I^2^**	**Cochran Q**	***p*** **-value**	**Number of Studies**	**Number of Patients**	**HR (95% CI)**	**I^2^**	**Cochran Q**	***p*** **-value**
Overall	7	124 501	0.919 (0.856–0.986)	88.50%	<0.0001	**0.0193**	7	323 854	0.898 (0.821–0.981)	87.20%	<0.0001	**0.0173**
PDI^b^	3	28 844	0.896 (0.762–1.05)	91.70%	<0.0001	0.184	2	211 585	0.881 (0.806–0.963)	34.20%	0.218	**0.0054**
Vegetarian Diet^c^	2	58 274	0.887 (0.782–1.01)	0.00%	0.639	0.0611	1	16 254	0.810 (0.721–0.911)	22.20%	0.257	**0.0004**
By Healthful vs. Unhealthful PDI						**0.05^a^**						0.18^a^
hPDI	3	52 770	0.912 (0.794–1.05)	92.10%	<0.0001	0.190	3	71 301	0.870 (0.800–0.946)	57.50%	0.0949	**0.0011**
uPDI	3	18 966	1.05 (1.01–1.09)	0.00%	0.384	**0.0123**	2	221 177	1.11 (0.787–1.56)	95.50%	<0.0001	0.555

*p-value < 0.05 is significant; HR, hazard ratio; CI, confidence interval; PDI, plant-based dietary index; hPDI, healthful plant-based dietary index; uPDI, unhealthful plant-based dietary index;*

a *Denotes p-value comparing subgroup difference between hPDI and uPDI;*

b *Highest quintile/tertile vs. lowest quintile/tertile;*

c *Vegetarians vs. regular meat eaters. Bolded values denote that p-value < 0.05*.

The cardiovascular benefits from plant-based diets have been attributed to a higher intake of fiber, plant protein, plant-based unsaturated fatty acids, phytochemicals (e.g., antioxidants and plant sterols), with a lower intake of saturated fat and energy density (53). Consequently, this reduces intermediate CVD risk factors, including low-density lipoprotein cholesterol, total cholesterol, blood pressure, and body weight (4, 54–57). Obesity has also been suggested to be a prominent CVD risk factor through adversely affecting plasma lipids, thus diminishing the cardioprotective effects of high-density lipoprotein cholesterol (58). Hence, the reduction of body fat through the consumption of plant-based diets may help to delay CVD initiation or progression (59, 60). Furthermore, the antiinflammatory and antithrombotic properties of the bioactive compounds found in fruits and vegetables may also confer cardioprotective effects (61–63). Thus, vegetarian diets have been proposed to ameliorate inflammatory processes that underlie the pathophysiology of atherosclerotic CVD (64–66).

Our results on an overall plant-based diet and lowered risk of CVD are broadly consistent with prior studies which found a lowered incidence of coronary or ischemic heart disease among vegetarians compared to non-vegetarians (15, 22). However, unlike previous studies (15, 22), greater adherence to plant-based diets was found to decrease cardiovascular mortality with the inclusion of more recent studies (42, 43, 46). In addition, this divergence may partly be explained by the differing definitions of plant-based diets where prior studies defined vegetarianism based on the complete exclusion of some or all animal foods (11–13). Comparatively, this review further examined gradations of adherence to a predominantly plant-based diet based on diet scores derived from PDIs (42–44, 46–49, 52). Importantly, these recently newly developed plant-based indices allow the quantification of synergistic effects of dietary compositions and hold wide applicability in the healthcare setting as recommendations of moderate dietary changes, such as gradual reductions in animal food intake, and are likely easier to adopt and adhere to than the complete exclusion of animal foods (53). However, adherence to an unhealthful plant-based diet was found to increase cardiovascular mortality. Those in the highest quintile of uPDI consumed foods with higher glycemic load and index, more added sugar, and lower levels of dietary fiber, unsaturated fats, micronutrients, and antioxidants, potentially contributing to poorer glycemic control (19, 67), lipid metabolism (68), and weight gain (69, 70). Particularly, consumption of sugar-sweetened beverages has been found to be positively associated with mortality primarily through CVD mortality, exhibiting a graded association with dose (71). Regardless, these findings should be interpreted with caution considering the small number of studies, and further investigation to confirm the impact of an unhealthful plant-based diet on cardiovascular mortality is warranted.

Current guidelines by the Academy of Nutrition and Dietetics (72), the American Dietetic Association (73), and most recently, the Dietary Guidelines for Americans 2015–2020 (74) recommend appropriately planned vegetarian diets for improved health. The key elements of the eating pattern include reduction in animal products, highly refined grains, added sugars, and oils, and consumption of an abundance of fresh vegetables, whole grains, and fruits. In key randomized clinical trials of plant-based diets, low-glycemic index foods were also found to improve cardiovascular risk factors (75). Besides emphasizing the quality of food in plant-based dietary patterns, guidelines should also develop appropriate resources and tools for healthcare professionals to effectively counsel their patients on plant-based nutrition therapy (76). Interestingly, plant-based diets have been shown to be as acceptable as other therapeutic diets, suggesting their suitability for long-term use (77–79). For instance, large healthcare organizations such as Kaiser Permanente have been successfully promoting vegetarian diets for patients as a cost-effective, low-risk solution that targets multiple chronic diseases simultaneously (80).

### Strengths and Limitations

*Hitherto*, plant-based diets have not been examined in randomized controlled trials of hard cardiovascular endpoints, possibly limited by the long induction periods of CVD with respect to its dietary etiology. This study, therefore, presents high quality evidence for major clinical endpoints such as cardiovascular mortality, CVD, and stroke by pooling a robust sample size of 410,085 participants in HR which remains the gold standard for time-dependent survival analysis. Most included studies were adjusted for important confounders including age, BMI, smoking, and alcohol history. In addition, dietary intake was largely assessed *via* the validated food frequency questionnaire which has been shown to have high reproducibility (81). However, several limitations should be accounted for when interpreting the study results. First, I^2^, a measure of heterogeneity, was significantly large in this analysis albeit attributable to the large sample size. It is well recognized that large sample sizes often inflate heterogeneity estimates (33, 34), as seen in a previous study involving a large cohort, resulting in an I^2^ > 90% (82). Most included studies originated from North America and Europe, except for one study from Asia. Given that plant-based dietary patterns are likely to vary across cultural contexts (83), more evidence is warranted to determine the cardiovascular effects of a plant-based diet within the Asian population. Further stratifications to examine the effects of gender and the duration of adherence to the plant-based pattern on cardiovascular health were also limited with the available data. Nevertheless, the beneficial effects on established proximal determinants of CVD in addition to the inverse association between plant-based dietary patterns and cardiovascular mortality in our pooled analysis lend strong support for the favorable role of quality plant-based foods in promoting cardiometabolic health.

## Conclusion

This study provides important evidence to suggest a possible protective role of plant-based dietary patterns against cardiovascular mortality and CVD among the general population. Importantly, not all plant foods are equally beneficial, but unhealthful refined carbohydrates, added sugars, and oils should be avoided. In future studies, it may be worth exploring the potential of healthful plant-based diets as secondary prevention among patients with preexisting CVD and the dose-response association between the level of plant food intake and cardiovascular benefits.

## Data Availability Statement

The original contributions presented in the study are included in the article/Supplementary Material, further inquiries can be directed to the corresponding authors.

## Author Contributions

JQ, GL, WL, CN, WS, JT, XP, and YC contributed to the acquisition of data, analysis and interpretation of data, and drafting of the article. JQ, GL, WL, and CN aided in formal analysis, methodology, and validation. MM, SC, NN, MC, RF, NC, and PL aided in revising the article critically for important intellectual content. All the authors read and gave final approval of the version to be submitted. The manuscript, including related data, figures and tables has not been previously published, and that the manuscript is not under consideration elsewhere.

## Conflict of Interest

The authors declare that the research was conducted in the absence of any commercial or financial relationships that could be construed as a potential conflict of interest.

## Publisher's Note

All claims expressed in this article are solely those of the authors and do not necessarily represent those of their affiliated organizations, or those of the publisher, the editors and the reviewers. Any product that may be evaluated in this article, or claim that may be made by its manufacturer, is not guaranteed or endorsed by the publisher.
