# Supplementary material for: The Association of Plant-Based Diet With Cardiovascular Disease and Mortality: A Meta-Analysis and Systematic Review of Prospect Cohort Studies
Source: Front Cardiovasc Med. 2021 Nov 5;8:756810. doi: 10.3389/fcvm.2021.756810 (PMC8604150; doi:10.3389/fcvm.2021.756810)
Supplement: Supplementary file 1 [file Data_Sheet_1.PDF]

S1 File Full Search Strategy

1. exp vegetarians/ or (plant* adj4 (diet* or nutri* or food* or eat* or consum*)).tw. or vegan*.tw.
2. exp Cardiovascular Diseases/ or (cardio* or cardia* or heart* or coronary* or angina* or ventric* or myocard* or pericard* or arrhythmi* or thrombo* or atrial next fibrillat* or mortality).tw.
3. exp Clinical Trial/ or randomized controlled trial.pt. or controlled clinical trial.pt. or randomi*.tw. or placebo.tw. or trial.ti or exp cohort studies/ or cohort$.tw. or controlled clinical trial.pt. or epidemiologic methods/
4. exp Stroke/ or (stroke or strokes or cerebrovasc* or apoplexy).tw.
5. 2 or 4
6. 1 and 3 and 5
7. 1 and 5
8. 3 and 7
