# Supplementary material for: The Association of Plant-Based Diet With Cardiovascular Disease and Mortality: A Meta-Analysis and Systematic Review of Prospect Cohort Studies
Source: Front Cardiovasc Med. 2021 Nov 5;8:756810. doi: 10.3389/fcvm.2021.756810 (PMC8604150; doi:10.3389/fcvm.2021.756810)

S2 File Funnel Plot for Hazard Ratio for Cardiovascular Mortality

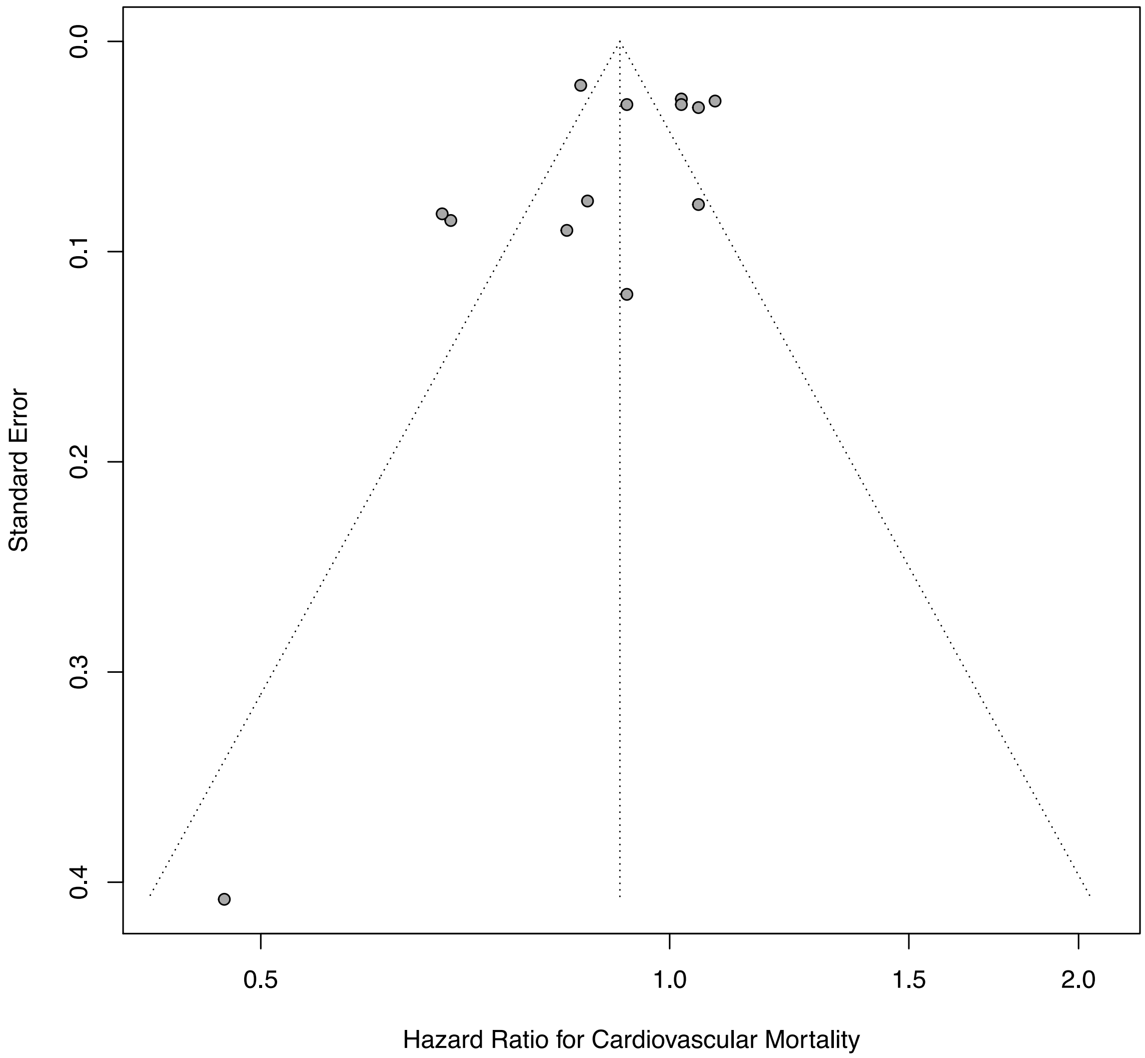

Funnel Plot for Hazard Ratio for Cardiovascular Disease (CVD)

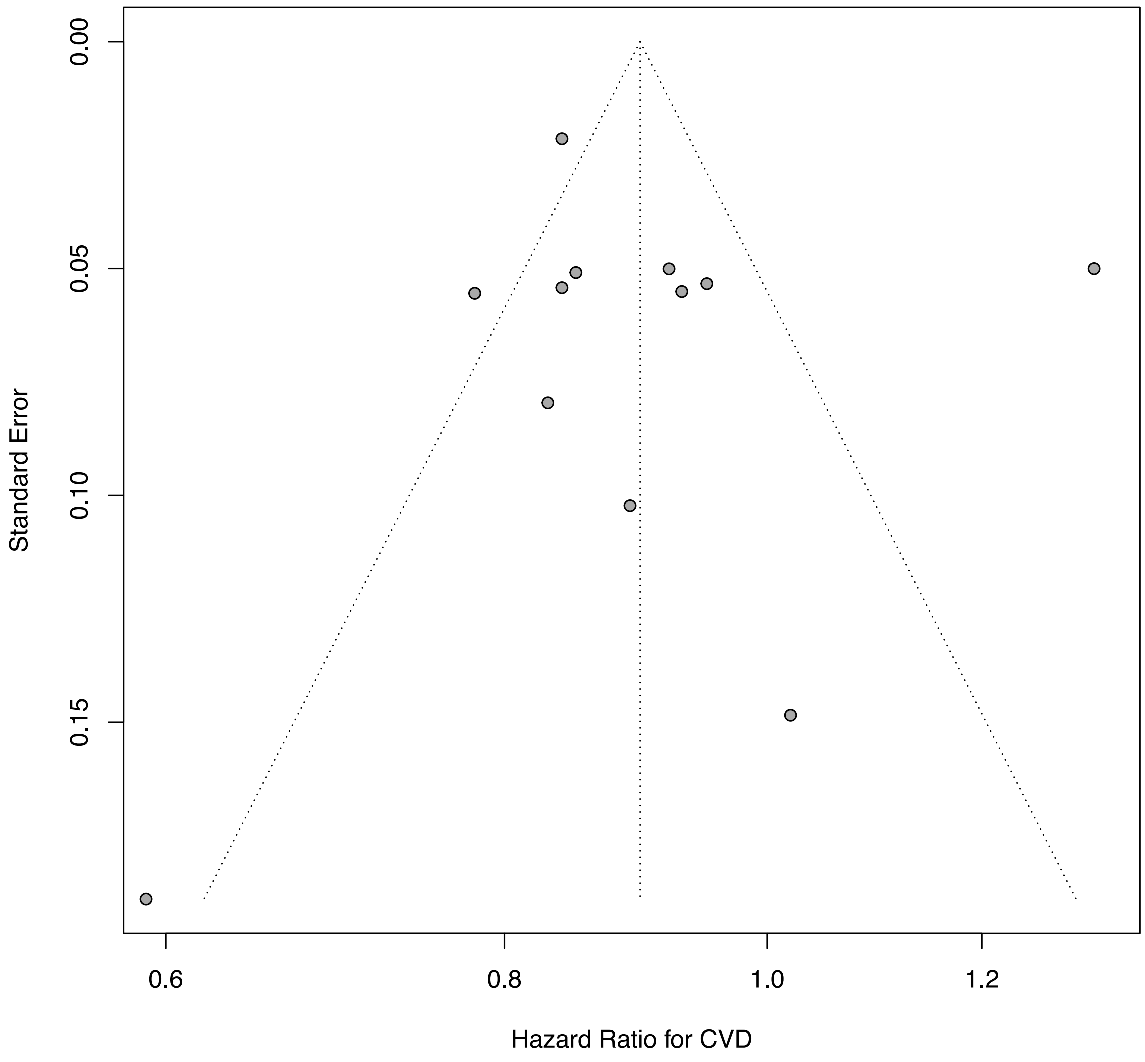

Supplement: Supplementary file 2 [file Data_Sheet_2.PDF]
